# Broader neutralization of CT-P27 against influenza A subtypes by combining two human monoclonal antibodies

**DOI:** 10.1371/journal.pone.0236172

**Published:** 2020-07-29

**Authors:** Kye Sook Yi, Jung-ah Choi, Pankyeom Kim, Dong-Kyun Ryu, Eunji Yang, Dain Son, JiYoung Shin, Hayan Park, Sena Lee, HyunJoo Lee, Bok-Hyeon Im, Ji-Sang Chae, Eun Beom Lee, Soo-Young Lee, Manki Song

**Affiliations:** 1 Biotechnology Research Institute, Celltrion Inc., Incheon, Republic of Korea; 2 Science Unit, International Vaccine Institute, Seoul, Republic of Korea; University of South Dakota, UNITED STATES

## Abstract

There are several broadly neutralizing monoclonal antibodies that neutralize influenza viruses with different mechanisms from traditional polyclonal antibodies induced by vaccination. CT149, which is one of the broadly neutralizing antibodies, was also previously reported to neutralize group 2 and some of group 1 influenza viruses (13 out of 13 tested group 2 viruses and 5 out of 11 group 1 viruses). In this study, we developed another antibody with the aim of compensating partial coverage of CT149 against group 1 influenza viruses. CT120 was screened among different antibody candidates and mixed with CT149. Importantly, although the binding sites of CT120 and CT149 are close to each other, the two antibodies do not interfere. The mixture of CT120 and CT149, which we named as CT-P27, showed broad efficacy by neutralizing 37 viruses from 11 different subtypes, of both group 1 and 2 influenza A viruses. Moreover, CT-P27 showed *in vivo* therapeutic efficacy, long prophylactic potency, and synergistic effect with oseltamivir in influenza virus-challenged mouse models. Our findings provide a novel therapeutic opportunity for more efficient treatment of influenza.

## Introduction

Seasonal influenza, an acute respiratory infection with global impact, is caused in humans primarily by circulating subtypes of influenza A viruses, which are the only ones known to cause pandemics. Influenza illness ranges from mild to severe and is responsible for significant numbers of hospitalizations and deaths globally. Annual influenza epidemics are estimated to result in about 3 to 5 million cases of severe illness, and approximately 290,000 to 650,000 deaths (https://www.who.int/news-room/fact-sheets/detail/influenza-(seasonal)) worldwide. In the developed countries, most deaths occur in patients >65 years of age [1], while in developing countries, influenza accounts for 99% of deaths related to lower respiratory tract infections in children <5 years of age [2]. Normally, influenza A viruses can be classified in group 1 and group 2, according to the phylogenetic relatedness. Group 1 influenza virus contains the pandemic H1 subtype, included in the seasonal influenza virus vaccine, as well as H2, H5, H6, and H9 subtypes. Another co-circulating seasonal influenza virus, H3 subtype, is classified as group 2 influenza virus along with H7 and H10 subtypes [3].

Two classes of antiviral drugs used against influenza are neuraminidase inhibitors (oseltamivir, zanamivir, and peramivir) and M2 protein inhibitors (amantadine derivatives). Neuraminidase inhibitors are currently preferred for influenza infections as they are less toxic and more effective compared to M2 protein inhibitors. Recently, another type of antiviral has been approved, baloxavir marboxil, which targets cap-dependent endonuclease. However, these antivirals induce resistant viruses quickly. Most influenza viruses acquire amantadine resistance, and the ratio of oseltamivir-resistant viruses has also been increasing [4, 5]. In the case of baloxavir, reduced susceptibility was reported in its clinical trials [6, 7].

Recently, broadly neutralizing monoclonal anti-influenza antibodies have been reported. Most of them bind on the conserved stem region in hemagglutinin (HA) protein, and they also inhibit HA-mediated viral fusion between endosomal and viral membranes. Some of them neutralize group 1 (C179, F10, 6F12 and CR6261), group 2 (CR8020, CR8043 and 9H10), or both group 1 and 2 (FI6, VIS410, 39.29, CT149 and MEDI8852) influenza A viruses [8–19]. These antibodies inspired development of universal influenza vaccines. Moreover, several of them, such as CR6261, CR8020, VIS410, and MEDI8852 are currently under clinical trials with the purpose of providing another influenza treatment option, especially for drug-resistant strains or high-risk populations.

Previously, we reported a broadly neutralizing anti-influenza antibody, CT149 [18], which could neutralize group 2 viruses and some of group 1 influenza viruses (13 out of 13 tested group 2 viruses and 5 out of 11 group 1 viruses) *in vitro*. CT149 could protect mice from A/California/04/2009 (H1N1) infection but could not neutralize it in microneutralization assay *in vitro*. Furthermore, it was suggested that CT149 shows *in vivo* efficacy through antibody-dependent cellular cytotoxicity (ADCC). Here, we developed another broadly neutralizing anti-influenza antibody aimed at complementing the narrow neutralization spectrum of CT149 against group 1 influenza viruses.

## Materials and methods

### Recombinant HA (rHA)

The following commercially available monomeric recombinant HAs expressed from both baculovirus and mammalian expression systems were utilized for the studies: A/California/04/2009 (H1N1) (Sino Biological, Beijing, China, Cat. 11055-V08H) and A/California/07/2009 (H1N1) (Immune Technology, NY, USA, Cat. IT-003-SW12ΔTMp). In addition, trimeric recombinant HAs, which have fold on trimerization sequences, were obtained from the Influenza Reagent Resource (https://www.internationalreagentresource.org/: A/California/04/2009 (Cat. FR-180)).

### Viruses and cells

Viruses used in this study comprised wild-type isolates and reassortants, containing internal genes from A/Puerto Rico/8/1934 or A/Ann Arbor/1960, which were developed as suitable candidate vaccine viruses for vaccine manufacturing. Viruses were propagated in Madin-Darby Canine Kidney (MDCK) cells or in embryonated eggs. All infectious wild-type H5N1 viruses were handled in Biosafety Level 3 facilities including enhancements required by the U.S. Department of Agriculture and the Select Agent Program [20].

Wild type A/Vietnam/1203/2004 (H5N1), mouse-adapted A/California/04/2009 (H1N1), and A/Anhui/1/2013 (H7N9) (AN/13) were amplified in embryonated eggs and mouse-adapted A/Hong Kong/1968 (H3N2) was propagated in MDCK cell cultures. Virus titers were determined by plaque assay in MDCK cells (plaque-forming units [PFU]/mL) or by endpoint dilution in MDCK cells (median tissue culture infectious dose [TCID_50_]) or eggs (egg infectious dose [EID_50_]/mL). All viruses were provided by Centers for Disease Control and Prevention (CDC, Atlanta, GA, USA).

MDCK (CCL-34) and Chinese Hamster Ovary (CHO-K1, CCL-61) cells were obtained from the American Type Culture Collection (ATCC, Manassas, VA) and cultured in Dulbecco’s Modified Eagle’s Medium (DMEM, Gibco, Cat. 11965) with or without 10% Fetal Bovine Serum (FBS).

### Transient and stable expression of antibodies

Antibody heavy and light chains were subcloned into the MarEx-based expression vector, pCT107 (patent, US 8772021 B2 (2006)). For transient expression, plasmids encoding antibodies of interest were transfected into F2N78 [21], harvested 5 days after transfection and purified by protein A affinity chromatography. Stable CHO cell lines were established to prepare large quantities of antibodies following methotrexate (MTX) selection (patent, US 8772021 B2 (2006)).

### Antibody screening

The monoclonal antibody pool, generated from convalescent patients infected with 2009 pandemic H1N1 strain, was screened again following the method used for screening CT149 [18]. All donors gave written informed consent for research use of blood samples following protocols approved by Institutional Review Board at Severance hospital in Yonsei university, Seoul, Korea (approval number: 4-2009-0461). Human single B cells that secret antibodies against HA (A/California/04/2009 (H1N1)) were selected and their antibody genes were isolated. After expressing the antibodies, they were screened again through HA ELISA (A/California/04/2009 (H1N1)) and microneutralization assay with several influenza viruses.

### ELISA

Monomeric (A/California/04/2009(H1N1) (Sino Biological, Beijing, China, Cat. 11055-V08H)) or trimeric rHA (A/California/04/2009 (Cat. FR-180)) were diluted in carbonate/bicarbonate coating buffer and added to 96-well microtiter plates (Nunc, Denmark, Cat. 449824) (250 ng/mL, 50 μL/well). After blocking with 1% bovine serum albumin (BSA) in PBS, antibodies (3-fold dilutions starting from 1 μg/mL) were added to each well and incubated at 22°C for 1 hour. After washing, horseradish peroxidase-conjugated goat anti-human gamma chain (Zymed, USA, Cat. 62.8420) was added and incubated at 22°C for 1 hour. After washing, the plate was incubated with tetramethylbenzydine (TMB; Sigma-Aldrich, MI, USA, Cat. T0440) and the incubation stopped by adding 1N HCl. The absorbance at 450/570 nm was recorded by a plate reader (Spectramax plus 384, Molecular Device) and the data was plotted with the Prism software package (GraphPad Software Inc. USA).

### Microneutralization (MN) assay

Microneutralization (MN) assay was performed as described previously [18] with some modifications. MDCK cells (3.0X104 cells/well) were prepared in 96-well plate and incubated for 24±0.5 hours at 37°C. 2-fold serially diluted antibodies were mixed with equal volume of 100 TCID_50_ viruses, and the mixture was then incubated in 96-well plates for 1 hour at 37°C. After 1hour, the mixture was added to the prepared cell plate and incubated for 72±1 hours at 37°C. Cultured supernatant was transfer to V bottom 96-well plate and reacted with chicken red blood cells (RBCs) for agglutination. After 30 min incubation at RT, agglutination of the RBCs were observed. For ELISA assay, MDCK cells were incubated with antibody-virus mixture for 24 hours and viral protein was detected with anti-NP antibody (Millipore, Germany, Cat. 18–152). The IC_50_ values of CT120, CT149, and CT-P27 were calculated with Reed-Muench method.

### Hemagglutination Inhibition (HI) assay

Viral antigens were mixed with 2-fold serially diluted antibodies in PBS, dispensed into 96-well plates, and incubated at 20–22°C for 30 min. Next, 0.5% suspension of turkey erythrocytes (Rockland Immunochemicals, Inc., Limerick, PA, USA, Cat. R408-0050) was added to each well, and the mixture was incubated for 30 min at 20–25°C before visual scoring for hemagglutination activity.

### Trypsin cleavage inhibition assay

One microgram of A/California/07/2009 (H1N1) (Immune Technology, NY, USA, Cat. IT-003-SW12ΔTMp) was pre-incubated in 50 μL PBS with 2 μg Fab at 37°C for 1 hour. After adding 1 ng of TPCK trypsin (Sigma, Louis, MO, Cat. T1426), the reaction was further incubated at 37°C for 30 min and then subjected to SDS-PAGE. HA0 and HA2 fragments were detected by western blot using mouse anti-His antibody (AbCam, MA, USA, Cat. AB15149).

### HA-expressing CHO cell lines

The complete coding regions of HA from A/California/04/2009 (H1N1), A/Japan/305/1957 (H2N2), A/Brisbane/10/2007 (H3N2), and A/Vietnam/1203/2004 (H5N1) were synthesized from sequences obtained from the National Center for Biotechnology Information (NCBI) database and cloned into pCT107 vector. HA expression plasmids were transfected into CHO-K1 cells using Lipofectamine^TM^ LTX Reagent (Invitrogen, Cat. 15338–100) following the manufacturer’s instructions. Transfected cells were selected with MTX, and those constitutively expressing HA were identified by immunofluorescence with FITC-conjugated subtype-specific anti-HA antibodies.

### Syncytia formation assay

H1, H2, H3, or H5 HA-expressing cells cultured in 6-well plates were treated for 5 min with 4 μg/mL TPCK trypsin (Sigma, Louis, MO, Cat. T1426) to cleave HA0 into HA1 and HA2, and the reaction was quenched by adding 0.3 mL of FBS (final concentration, 10%). Antibodies were added (20 μg/mL) to the wells and incubated at 37°C for 1 hour. After washing with PBS, cells were exposed to pre-warmed, low-pH buffer (150 mM NaCl, 10 mM HEPES, pH 5.0) and incubated at 37°C for 6 min. The acidic medium was replaced with DMEM supplemented with 10% FBS and cells were incubated for 1 hour. Cells were fixed with ice-cold methanol, then stained with trypan blue. Syncytia formation was evaluated qualitatively by observing random fields under an inverted microscope (Nikon Eclipse TS100) and photographed using a Digital color CCD camera.

### Protection of mice from influenza virus infection

Six- to nine-week-old female BALB/c mice were purchased from Orient Bio (Korea) and Semtako (Korea). Before the experiment, animals were acclimated for 7 days and housed in the pathogen-free facility located at the International Vaccine Institute, Seoul, Korea. This study was carried out in accordance with the recommendations in the Guidelines for Humane Endpoint Criteria of International Vaccine Institute and Korean Animal Welfare Act and Laboratory Animal Act. All animal studies for the evaluation of the protective effects of CT120, CT149, and CT-P27 against influenza viruses were conducted in compliance with Institutional Animal Care and Use Committee-approved protocols of International Vaccine Institute (approval number for International Vaccine Institute (IVI) is 2012–011 and 2015–011). A/California/04/09 (H1N1) and A/Philippines/2/1982 (H3N2) virus infection was performed under ketamine solution anesthesia, and all efforts were made to minimize suffering. The body weight and survival rate of the mice was monitored daily for 14 or 15 days after virus infection. To calculate survival rates, the criteria for determining dead mice were either actual death or displaying 30% or greater reduction in body-weight loss; in the latter case, mice were subsequently euthanized using carbon dioxide on the day they found the weight loss of more than 30%. CT-P6, a non-relevant isotype IgG1, was injected as the negative control for all studies. All animal experiments were conducted by the authorized and experienced researchers.

To evaluate therapeutic efficacy of CT-P27 against mouse-adapted H1N1 (A/California/04/2009), 10 mice per group were anesthetized and inoculated with 5 LD_50_ (median lethal dose) of virus diluted in 50 μL of PBS. Twenty-four hours post-infection, treatment groups were intraperitoneally injected with one of the three antibody doses (7.5, 15, or 30 mg/kg). A similarly designed experiment was also performed against mouse-adapted H3N2 (A/Philippines/2/1982).

To evaluate prophylactic efficacy of CT-P27 against mouse-adapted H1N1 (A/California/04/2009) or mouse-adapted H3N2 (A/Philippines/2/1982), 10 mice per group were anesthetized and inoculated with 5 LD_50_ of virus diluted in 50 μL of PBS, Treatment groups were intraperitoneally injected with 30 mg/kg of CT-P27 antibody 14, 9, 5, 3, or 1 day(s) before infection.

To evaluate combined efficacy of CT-P27 with oseltamivir against mouse-adapted H1N1 (A/California/04/2009), 10 mice per group were anesthetized and inoculated with 3 LD_50_ of virus diluted in 50 μL of PBS. Twenty-four hours post-infection, oseltamivir treatment groups were orally administered two different doses (20 or 100 mg/kg) twice daily for 5 days. CT-P27 treatment groups were intraperitoneally injected with one of the three antibody doses (1.875, 3.75, or 7.5 mg/kg). The combination treatment groups were administered oseltamivir 20 mg/kg/day for 5 days with a single dose of CT-P27 (1.875 or 3.75 mg/kg).

To evaluate Fc-related efficacy of CT-P27 against mouse-adapted H1N1 (A/California/04/2009), 10 mice per group were anesthetized and inoculated with 2 LD_50_ of virus diluted in 50 μL of PBS. Twenty-four hours post-infection, treatment groups were intraperitoneally injected with one of the three antibody doses (3, 4, or 8 mg/kg) of CT120 or CT120 LALA mutant The experiment with CT149 and CT149 LALA mutant was also performed against mouse-adapted H3N2 (A/Philippines/2/1982). Treatment groups were intraperitoneally injected with one of the three antibody doses (12, 16, or 20 mg/kg) of CT149 or CT149 LALA mutant.

### Egress assay

Twenty-four hours before virus infection, MDCK cells (2x10^4^ cells/well) were seeded in 96-well plates (Corning, US, Cat. 3595) with complete media containing 10% FBS (Gibco, US, Cat. 10099–141) in DMEM (Gibco, US, Cat. 11885–084). The viruses (A/Ohio/83 or A/Philippines/2/82) were diluted at 1 multiplicity of infection (MOI) with OptiPRO media (Gibco, US, Cat. 12309–019). Following two washes with PBS, the viral inoculum was added and incubated at 37°C in CO_2_ incubator for 4 hours to allow virus entry (assay plate). CT120, CT149, CT-P27, and negative control (CT-P6) were 4-fold serially diluted from 400 to 0.024 μg/mL with complete media in 96-well plates (dilution plate). In parallel, 10 mM of oseltamivir was prepared as positive control, ranging from 40 to 0.002 μM. After washing to remove input viruses, the prepared antibodies and oseltamivir in dilution plate were added to the infected MDCK cells in the assay plate, in duplicates. The assay plates were incubated in CO_2_ incubator at 37°C overnight. After incubation, virus titer was assessed by using HA assay with 0.5% chicken RBC.

## Results

### Combination of two monoclonal anti-influenza antibodies

Although previously screened anti-influenza antibody, CT149, protects mice from H1N1, H3N2, and H5N1 infection, it could not efficiently neutralize group 1 viruses *in vitro*. Therefore, we planned to screen another broadly neutralizing antibody, especially potent against group 1 influenza viruses, and mix it to CT149 to compensate for its potency.

CT120 was selected among hundreds of antibody candidates. Although CT120 could not neutralize group 2 viruses, it could neutralize group 1 viruses efficiently (Table 1). Amino acid analysis revealed that heavy and light chains of CT120 have high similarity with IgHV1-69*01 and IGKV3-15*01, respectively.

**Table 1:** *In vitro* neutralization activity of CT-P27 against influenza A viruses from diverse subtypes.

Group	Subtype	Virus	IC_50_ (μg/mL)		
			CT120	CT149	CT-P27
1	H1N1	A/PuertoRico/8/1934	1.17	N^1^	1.30
		A/Texas/05/2009-RG15	0.59	N^1^	2.93
		A/New Caledonia/20/1999	1.30	N^1^	1.46
		A/Solomon Islands/2006	0.39	N^1^	0.49
		A/Ohio/1983	0.10	N^1^	0.41
		A/California/04/2009 (mouse adapted)	1.30	N^1^	1.46
		A/California/04/2009 (wild)	0.33	>50	0.73
		A/Gwangju/61/2010, H275Y^4^	1.04	N^1^	1.63
		A/Ohio/07/2009	0.65	>100	1.46
	H2N2	A/Ann Arbor/6/1960 CA	0.78	N^1^	1.46
	H5N1	A/Vietnam/1203/2004 (VNH5N1-PR8/CDC-RG)	0.48	8.33	0.49
		A/Anhui/10/2005(H5N1)-PR8-IBCDC-RG6	0.65	2.60	0.81
	H6N1^3^	A/EM/Korea/w340/2008	4.69	>50	6.51
	H6N2^3^	A/EM/Korea/w395/2010	4.69	>50	7.81
	H8N4^3^	A/EM/Korea/w141/2006	2.34	>50	3.26
	H8N8^3^	A/EM/Korea/w332/2008	1.30	>50	2.93
	H9N2	A/ck/HK/G9/1997(H9N2)/PR8-IBCDC-2	2.08	6.25	1.95
	H12N5^3^	A/EM/Korea/w373/2008	25.00	>50	23.44
	H12N7^3^	A/EM/Korea/w424/2012	20.83	>50	23.44
2	H3N2	A/HongKong/01/1968 (mouse adapted)	N^1^	1.56	2.93
		A/HongKong/01/1968 (wild)	N^1^	1.04	1.30
		A/Philippines/2/1982 (mouse adapted)	N^1^	0.59	0.73
		A/Sydney/5/1997	N^1^	1.30	3.91
		A/Beijing/32/1992-R-H3N2 PR8 reassortant	N^1^	2.34	2.93
		A/Moscow/10/1999	N^1^	2.34	2.93
		A/Perth/16/2009	N^1^	3.85	7.19
		A/Brisbane/10/2007	N^1^	1.17	1.46
		A/Switzerland/9715293/2013	NT^2^	4.69	6.35
		A/Hong Kong/485197/2014	N^1^	8.33	15.63
	H4N2^3^	A/EM/Korea/w398/2010	>50	5.21	5.86
	H4N6^3^	A/EM/Korea/w360/2008	>50	4.69	15.63
	H7N9	A/Anhui/1/2013	NT^2^	0.90	2.38
		A/Shanghai/2/2013	NT^2^	1.17	3.55
		A/Shanghai/1/2013	NT^2^	7.71	13.14
		A/Shanghai/1/2013, R292K^4^	N^1^	14.87	16.28
	H10N4^3^	A/EM/Korea/296/2007	>50	6.25	13.02
	H10N6^3^	A/EM/Korea/w333/2008	>50	5.21	11.72

Red: <5 μg/mL. Orange: 5–25 μg/mL. Yellow: 25–100 μg/mL. Green: Not neutralize.

^1^N: No neutralization effect: no response across antibody concentration.

^2^NT: Not tested.

^3^The influenza virus was isolated from swine.

^4^Oseltamivir-resistant virus

### IC_50_ Values

To test whether cotreatment with CT120 and CT149 can protect influenza virus infection broadly, the two antibodies were mixed in 1:1 ratio (w/w) and their efficacy was tested. In MN assay, CT120 showed neutralization activity in all tested group 1 influenza viruses (H1N1, H2N2, H5N1, H6N1, H8N8, H9N2, and H12H7) and CT149 neutralized part of group 1 (H5N1, H9N2) and all tested group 2 (H3N2, H4N6, H7N9, H10N6) viruses (Table 1). As expected, the 1:1 mixture of CT120 and CT149, which is named CT-P27, protected simultaneously against type 1 and type 2 influenza virus infection (Table 1).

### No interference between CT120 and CT149 in CT-P27

Most broadly neutralizing anti-influenza antibodies bind on the stem region of HA. They cannot inhibit hemagglutination, but protect HA cleavage and prevent syncytia formation. As expected, CT120 could not inhibit the hemagglutination of avian erythrocytes induced by A/Ohio/83 (H1N1) and A/Philippines/2/82 (H3N2) even at 80 μg/mL concentration (S1 Table). CT120 inhibited HA (A/California/04/2009 (H1N1)) cleavage by trypsin (S1 Fig) and low-pH induced syncytia formation of CHO cells expressing group 1 HAs on the surface (A/California/04/2009 (H1N1), A/Japan/305/1957 (H2N2) and A/Vietnam/1203/2004 (H5N1)), but not H3 (A/Brisbane/10/2007 (H3N2)) (S2 Fig). Despite the low resolution of X-ray crystallography results of CT120 with HA (A/Vietnam/1203/2004(H5N1)), we could determine the epitope sites of CT120 on stem region (S3 Fig, [22]. We found that the binding site of CT120 was similar to other influenza antibodies, such as F10 [9], but quite different from CT149, although they are slightly overlapping [18].

Therefore, because their epitope sites are close, possible interference between CT120 and CT149 before mixing them to extend strain coverage was a concern. The interference effect was thus monitored by measuring neutralization efficacy of one antibody after adding excess amount of the opposite antibody. As shown in Table 2, IC_50_ values of CT120 against A/Ohio/07/2009 (H1N1) mixed with various concentrations of CT149 (1:1, 1:2, 1:4, and 1:10 ratio of CT120 to CT149) were 0.59, 0.59, 0.29, and 0.33 μg/mL, respectively. IC_50_ values of CT149 against A/Philippine/2/1982 (H3N2) mixed with various concentrations of CT120 (1:1, 1:2, 1:4, and 1:10 ratio of CT149 to CT120) were 0.16, 0.20, 0.20, and 0.15 μg/mL. All of the IC_50_ values were within assay variation consistently. These results suggest that combination of CT120 and CT149 does not impact on their neutralizing effects against various influenza viruses. This was also reflected in IC_50_ values of CT-P27. Considering that CT-P27 is a 1:1 mixture of CT120 and CT149 and only half of the amount of CT-P27 is the amount of one antibody, IC_50_ values of CT-P27 follow the IC_50_ value of effective antibody component without interference from non-effective one (Table 1).

**Table 2:** IC_50_ values of CT120 and CT149 in the presence of molar excess of counter antibody.

**A/Ohio/07/2009 (H1N1)**	**Back titration**	**Ratio**	**MN assay result**
		**CT120: CT149**	**IC**_**50**_ **(CT120)**
	logTCID_50_/mL	1 = 100 μg/mL	μg/mL
	**3.33**	1:1	0.59
		1:2	0.59
		1:4	0.29
		1:10	0.33
**A/Philippine/2/1982 (H3N2)**	**Back titration**	**Ratio**	**MN assay result**
		**CT149: CT120**	**IC**_**50**_ **(CT149)**
	logTCID_50_/mL	1 = 100 μg/mL	μg/mL
	**3.13**	1:1	0.16
		1:2	0.20
		1:4	0.20
		1:10	0.15

Data represent mean values of results from three independent experiments.

### CT-P27 has broad therapeutic and prophylactic efficacy

After confirming that CT120 and CT149 can be combined as CT-P27 without interference *in vitro*, therapeutic and prophylactic efficacy of CT-P27 were evaluated *in vivo* in mouse models with influenza viruses from different groups.

**Fig 1:**
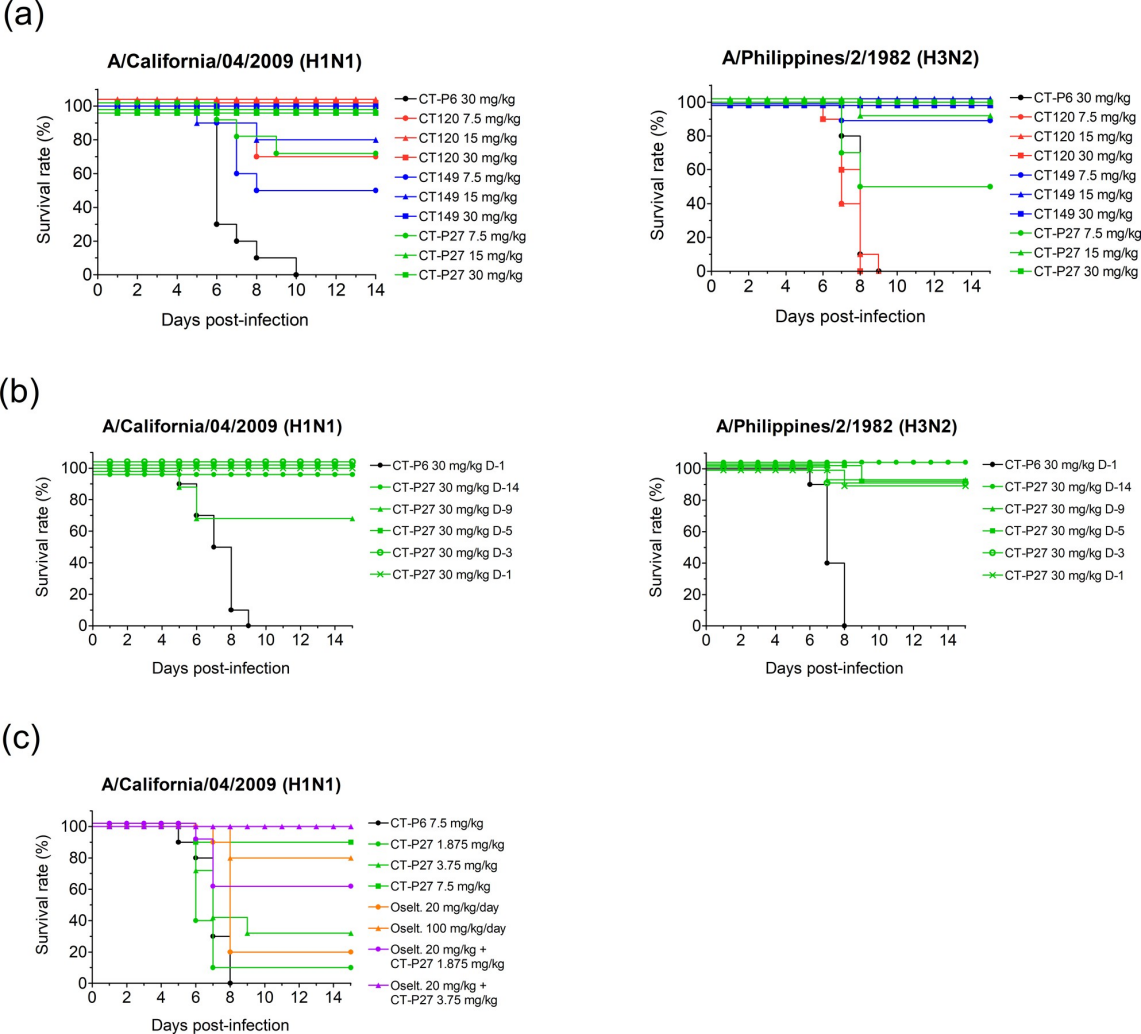
*In vivo* efficacy of CT-P27. Therapeutic and prophylactic efficacy of CT-P27 was observed in mice infected with a lethal dose of mouse-adapted A/California/04/2009 (H1N1) or A/Philippines/2/1982 (H3N2). Antibodies were administered 1 day after infection (A) or 14 to 1 day before virus challenge (B) and survival endpoints were monitored. Co-treatment effect of CT-P27 with oseltamivir was also investigated in mice. One day after challenging mouse-adapted A/California/04/2009 (H1N1), CT-P27 was administered once through intraperitoneal injection, with or without oral administration of oseltamivir for 5 days (C). This is a representative data from 2 repeated experiments.

To determine therapeutic activity of CT-P27, mice were infected with mouse-adapted A/California/04/2009 (H1N1) or mouse-adapted A/Philippines/2/1982 (H3N2). After 24 hours of infection, mice were treated with three different concentrations of CT120, CT149, or CT-P27 via intraperitoneal injection. In our previous study [18], CT149 could not protect mice from H1N1 influenza virus at low concentrations. Only 50% of mice were protected in the 7.5 mg/kg CT149-treated group. However, CT120 showed 100% protection against H1N1 virus in >15 mg/kg-treated group and 70% in 7.5 mg/kg-treated group. By adding CT120 to CT149, CT-P27 showed full protection in >15 mg/kg-treated group. This is an additive result from 7.5 mg/kg of CT149 (50% protection) and 7.5 mg/kg of CT120 (70% protection) (Fig 1A). Although CT120 could not protect mice from H3N2 infection, it showed no interference with CT149 efficacy in CT-P27-treated groups. Ninety percent of mice remained healthy in 15 mg/kg CT-P27-treated group, which was the same as in 7.5 mg/kg CT149-treated group (Fig 1A).

To study prophylactic efficacy of CT-P27 against A/California/04/2009 (H1N1) and A/Philippines/2/1982 (H3N2), 30 mg/kg of CT-P27 was administered at 14, 9, 5, 3, or 1 day(s) before the virus infection. CT-P27 showed 100% protection against H1N1 influenza virus challenge in each treated group except the group treated with CT-P27 9 days prior to virus infection (Fig 1). CT-P27 could also demonstrate its prophylactic effect against H3N2 virus with equal to or higher than 90% survival rate in each group (Fig 1B). According to these results, there is a strong evidence that CT-P27 has both therapeutic and prophylactic efficacy against group 1 and group 2 influenza viruses.

Since oseltamivir treatment within 48 hours after symptom onset is a common strategy for influenza infection, combined effect of CT-P27 with oseltamivir treatment was also monitored. Mice were infected with 3 LD_50_ of mouse-adapted A/California/04/2009 (H1N1) and treated with CT-P27 as single intraperitoneal injection, oseltamivir for 5 days orally, or both drugs in combination 24 hours post-infection. Suboptimal concentration of CT-P27 which showed less than 100% survival rate were co-treated with oseltamivir to discriminate whether the improved survival rate was due to the co-treatment of oseltamivir or not. The groups treated with 1.875, 3.75, or 7.5 mg/kg of CT-P27 showed 10%, 30%, or 90% survival rate, respectively. However, when 20 mg/kg/day of oseltamivir was administered with 1.875 or 3.75 mg/kg of CT-P27, the survival rates increased up to 60 or 100%, respectively. These results suggest that co-treatment of CT-P27 and oseltamivir has synergistic effect and they can be co-administered for better treatment results (Fig 1C).

### CT-P27 prevents virus propagation through Fc function and egress inhibition

**Fig 2:**
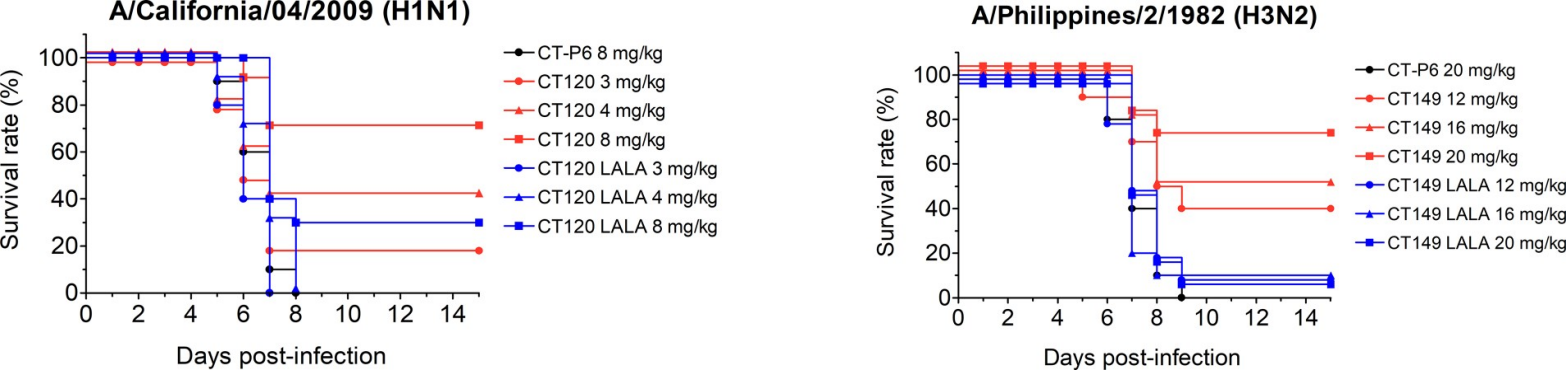
Fc function for *in vivo* efficacy of CT-P27. Mice were challenged with mouse-adapted A/California/04/2009 (H1N1) or A/Philippines/2/1982 (H3N2), and CT120, CT149, or double mutant was administered intraperitoneally after 24 hours. The survival rates were monitored for 15 days. This is a representative data from 2 repeated experiments.

Fc-mediated function was also confirmed as another mode of action of CT-P27 in addition to its viral neutralization effect. Previously, it has been reported that one *in vivo* mode of action of CT149 is ADCC. This was further confirmed for CT120 after introducing double mutation in Fc region (L234A and L235A) which is known to reduce ADCC and complement-dependent cytotoxicity (CDC) [23]. Mice were treated with suboptimal concentrations of antibodies 24 hours after infection with 2 LD_50_ of mouse-adapted A/California/04/2009 (H1N1) or A/Philippines/2/1982 (H3N2). To evaluate the Fc-related function of CT-P27 against the influenza virus, The dose levels of antibodies were below the saturation point of survival rate because if the dose level of each antibodies are too high, saturation effect will be induced by Fab-related function, regardless of Fc-related function. The survival rates in groups treated with a single dose of 3, 4, or 8 mg/kg CT120 were 20, 40, or 70%, respectively, while in groups treated with same dosage regimen of CT120 LALA mutant (L234A and L235A) they were 0, 0, or 30%, respectively. In addition, the survival rate in groups treated with a single dose of 12, 16, or 20 mg/kg CT149 were 40, 50, or 70%, respectively, while in groups treated with same dosage regimen of CT149 LALA mutant (L234A and L235A) they were 10% for all doses (Fig 2). These results suggest that Fc-mediated immune function plays an important role in anti-influenza activity of CT-P27 *in vivo* in terms of survival rate. Simultaneously, these reveal that the antigen recognizing the Fab region also participates in *in vivo* efficacy since LALA mutants (L234A and L235A) can protect mice from the viral challenge.

Recently, two studies have suggested that some anti-influenza antibodies can inhibit virus egress via binding to HA on the surface of the infected cells. Brandenburg *et al*. showed that HA head-specific antibody reduced the viral particles released from infected cells, while HA stem-specific antibody had no effect on virus release from the cells [24]. By contrast, Tan *et al*. revealed that HA stalk-targeting antibody led to decreased viral egress from the infected cells [14]. To address whether CT120, CT149, and CT-P27 could inhibit the egress step in the virus life cycle, *in vitro* egress assay was performed. MDCK cells were infected with A/Ohio/1983 (H1N1) or A/Philippines/2/1982 (H3N2). The infected cells were incubated to produce progeny viruses in the presence of CT120, CT149, or CT-P27. Released viruses were measured by HA assay since CT120, CT149, and CT-P27 could not inhibit hemagglutination even at 1.2 mg/mL concentration. The results show that CT120 and CT149 inhibited egress of H1N1 and H3N2 viruses, respectively, and CT-P27 inhibited egress of both H1N1 and H3N2 viruses (Table 3).

**Table 3:** *In vitro* egress assay in the presence of CT120, CT149 and CT-P27.

Antibody (μg/ml)		0.02	0.10	0.39	1.56	6.25	25	100	400
Oseltamivir (μM)		0.002	0.010	0.039	0.156	0.625	2.5	10	40
No antibody		+	+	+	+	+	+	+	+
CT-P6		+	+	+	+	+	+	+	+
Oseltamivir		-	-	-	-	-	-	-	-
H1N1	CT120	+	-	-	-	-	-	-	-
	CT149	+	+	+	+	+	+	+	+
	CT-P27	+	+	-	-	-	-	-	-
H3N2	CT120	+	+	+	+	+	+	+	+
	CT149	+	+	-	-	-	-	-	-
	CT-P27	+	+	+	-	-	-	-	-

+: Hemagglutination (= No egress inhibition)

-: No hemagglutination (= Egress inhibition)

MDCK cells were infected with viruses as indicated and incubated to allow virus entry only. After removal of input viruses, cells were treated with antibodies at different concentrations, along with oseltamivir as positive control and CT-P6, which is an unrelated IgG1, as negative control. The supernatants were harvested 24 hours later and analyzed by HA assay to measure the levels of released viruses from infected cells in the presence of antibodies. This is a representative data from 4 repeated experiments.

## Discussion

In this study, a novel broadly neutralizing anti-influenza antibody specific for group 1 viruses has been developed and mixed with CT149 to broaden its protection spectrum. CT-P27, a mixture of CT120 and CT149, has shown broader neutralizing effects *in vitro* and *in vivo* and its two components have shown no interference between them. CT-P27 has various modes of action, such as inhibiting infection, ADCC, and preventing viral egress. CT-P27 could use all modes of action, thus compensating neutralizing coverage of CT149 to group 1 viruses. In this study, CT-P27 has shown therapeutic effect, prophylactic efficacy, and synergistic effect when it was co-administered with oseltamivir.

We previously reported a broadly neutralizing anti-influenza antibody, CT149. Although it has shown therapeutic effect in mice against group 1 (A/California/04/2009 (H1N1) and A/Vietnam/1203/2004 (H5N1)) and group 2 (A/Hong Kong/1/1968 (H3N2) and A/Anhui/1/2013 (H7N9)) influenza A viruses, it could neutralize only group 2 influenza A viruses and some of group 1 viruses in MN assay. Several studies have reported that antibodies can inhibit various stages of the influenza life cycle. CT-P27 cannot inhibit receptor binding of influenza virus but can inhibit virus internalization by interrupting HA-mediated membrane fusion. However, CT149 alone cannot inhibit membrane fusion of certain subtypes (H2N2) [18], or neutralize several group 1 subtypes (Table 1). CT149 has shown *in vivo* efficacy by binding to HA and mediating ADCC [18]. However, there is a possibility that CT149 can lose binding capacity and fail to induce ADCC in a certain strain. The binding affinities of CT149 to HAs from different strains within group 1 are different and some are indeed quite low [18]. Therefore, prior to going further with CT149, we aimed to compensate its potency by adding another broadly neutralizing antibody especially effective to group 1 influenza viruses. Among candidate antibodies, CT120 was selected and showed the strongest efficacy against group 1 influenza A viruses. CT120 and CT149 were then mixed with the purpose of covering against all influenza A viruses. The resulting product, CT-P27, showed efficacy to all influenza A viruses through inhibiting infection, ADCC, and viral egress.

CT-P27 seems to be less effective than CT120 or CT149 in potency studies. However, since CT-P27 is a 1:1 mixture of CT120 and CT149, 30 mg of CT-P27 is equivalent to 15 mg of CT120 plus 15 mg of CT149. For example, 15 mg/kg of CT-P27 showed 90% protection rate and 7.5 mg/kg of CT149 showed the same protection rate when measuring survival in mice challenged with A/Philippines/2/1982 (H3N2). In this sense, 15 mg/kg of CT-P27 should be compared with 7.5 mg/kg CT120 and CT149 in A/California/04/2009 (H1N1)-challenged mice. Moreover, 15 mg/kg of CT-P27 conferred 100% protection rate while 7.5 mg/kg of CT120 and CT149 protected 70% and only 50%, respectively. From these experiments, the efficacy of CT-P27 was confirmed as the efficacy of CT120 plus CT149, and this result can be also applied to MN assay.

Due to its biological nature and lack of drug interference, CT-P27 could be beneficial for co-administration with other anti-influenza drugs that are already approved. Although CT-P27 may be administrated in high concentration because it contains half of effective antibodies against certain influenza strains. Many influenza experts believe that a combination of direct anti-influenza monoclonal antibodies with existing neuraminidase inhibitors will enhance the therapeutic effectiveness of the treatment and potentially reduce the possibility of selecting resistant strains. As shown in MN results, CT-P27 can neutralize oseltamivir-resistant viruses (A/Gwangju/61/2010 (H1N1, H275Y) and A/Shanghai/1/2013 (H7N9, R292K)) (Table 1). Therefore, co-treatment with CT-P27 and oseltamivir may neutralize oseltamivir-resistant viruses. CT-P27 is currently under clinical trials and we are studying more about the escape mutant generation under CT-P27 pressure.

## Supporting information

S1 Table Hemagglutination inhibition by CT-P27. (a) Hemagglutination inhibition assay was performed with H1N1 (A/Ohio/83) in the absence or presence of CT-P27. Monoclonal antibodies (CT-P27, CT-120, and CT-149) were two fold serially diluted from 80 μg/ml. Those antibodies were mixed with viruses and then RBC was added to allow hemagglutination. (+) and (-) represent hemagglutination and hemagglutination inhibition, respectively. NIBSC serum was used as positive control. b) Hemaggluination inhibition assay was carried out as described above except H3N2 (A/Philippines/2/82).
(DOCX)

S1 Fig Trypsin cleavage inhibition by CT120. Purified HA (A/California/04/2009) and trypsin were incubated with or without Fab and subjected to SDS-PAGE. HA0 and HA2 were detected with anti-His antibody. CT120 Fab inhibited cleavage of HA0 to HA1 and HA2 by trypsin, while non-relevant Fab, CT-P15, could not. This is a representative data from 3 repeated experiments.
(TIF)

S2 Fig Low-pH induced syncytia formation assay. CHO cells expressing HAs from A/California/04/2009 (H1N1), A/Japan/305/1957 (H2N2), A/Brisbane/10/2007 (H3N2), and A/Vietnam/1203/2004 (H5N1) were exposed to low-pH buffer in the presence of CT149 or an isotype-matched negative control antibody (CT-P6). Representative microscope fields were captured with a digital camera using an objective (10x). This is a representative data from 3 repeated experiments.
(TIF)

S3 Fig Epitope site of CT120. (a) The epitope site of CT120 on H5 (A/Vietnam/1203/2004(H5N1)) is displayed in a ribbon diagram. Pink and light blue colors represent HA1 and HA2 domain respectively and gray color is neighboring monomers. Filled spaces are epitope site of CT120. It is placed on stem region. (b) Epitope sites of F10, CT120, and CT149 is marked as pink color in space-filling model of corresponding HA structure. Yellow, light blue and gray colors are each monomers of trimeric HA.
(PPTX)
