# Supplementary material for: Broader neutralization of CT-P27 against influenza A subtypes by combining two human monoclonal antibodies
Source: PLoS One. 2020 Jul 29;15(7):e0236172. doi: 10.1371/journal.pone.0236172 (PMC7390384; doi:10.1371/journal.pone.0236172)
Supplement: S1 Table — (a) Hemagglutination inhibition assay was performed with H1N1 (A/Ohio/83) in the absence or presence of CT-P27. Monoclonal antibodies (CT-P27, CT-120, and CT-149) were two fold serially diluted from 80 μg/ml. Those antibodies were mixed with viruses and then RBC was added to allow hemagglutination. (+) and (-) represent hemagglutination and hemagglutination inhibition, respectively. NIBSC serum was used as positive control. b) Hemaggluination inhibition assay was carried out as described above except H3N2 (A/Philippines/2/82). (DOCX) [file pone.0236172.s001.docx]

**S1 Table. Hemagglutination inhibition by CT-P27** (a) Hemagglutination inhibition assay was performed with H1N1 (A/Ohio/83) in the absence or presence of CT-P27. Monoclonal antibodies (CT-P27, CT-120, and CT-149) were two fold serially diluted from 80 μg/ml. Those antibodies were mixed with viruses and then RBC was added to allow hemagglutination. (+) and (-) represent hemagglutination and hemagglutination inhibition, respectively. NIBSC serum was used as positive control. b) Hemaggluination inhibition assay was carried out as described above except H3N2 (A/Philippines/2/82).

(a)

| **Serum (%)** | 10 | 5 | 2.5 | 1.25 | 0.625 | 0.313 | 0.156 | 0.078 | 0.039 |
| --- | --- | --- | --- | --- | --- | --- | --- | --- | --- |
| **Antibody (μg/ml)** | 80 | 40 | 20 | 10 | 5 | 2.5 | 1.25 | 0.63 | 0.31 |
| **A/Ohio/83** | + | + | + | + | + | + | + | + | + |
| **NIBSC serum** | - | - | + | + | + | + | + | + | + |
| **CT-P27** | + | + | + | + | + | + | + | + | + |
| **CT-120** | + | + | + | + | + | + | + | + | + |
| **CT-149** | + | + | + | + | + | + | + | + | + |

(b)

| **Serum (%)** | 10 | 5 | 2.5 | 1.25 | 0.625 | 0.313 | 0.156 | 0.078 | 0.039 |
| --- | --- | --- | --- | --- | --- | --- | --- | --- | --- |
| **Antibody (μg/ml)** | 80 | 40 | 20 | 10 | 5 | 2.5 | 1.25 | 0.63 | 0.31 |
| **A/Philippines/2/82** | + | + | + | + | + | + | + | + | + |
| **NIBSC serum** | - | - | - | - | + | + | + | + | + |
| **CT-P27** | + | + | + | + | + | + | + | + | + |
| **CT-149** | + | + | + | + | + | + | + | + | + |
| **CT-120** | + | + | + | + | + | + | + | + | + |
