# Supplementary figures and images for: Broader neutralization of CT-P27 against influenza A subtypes by combining two human monoclonal antibodies
Source: PLoS One. 2020 Jul 29;15(7):e0236172. doi: 10.1371/journal.pone.0236172 (PMC7390384; doi:10.1371/journal.pone.0236172)

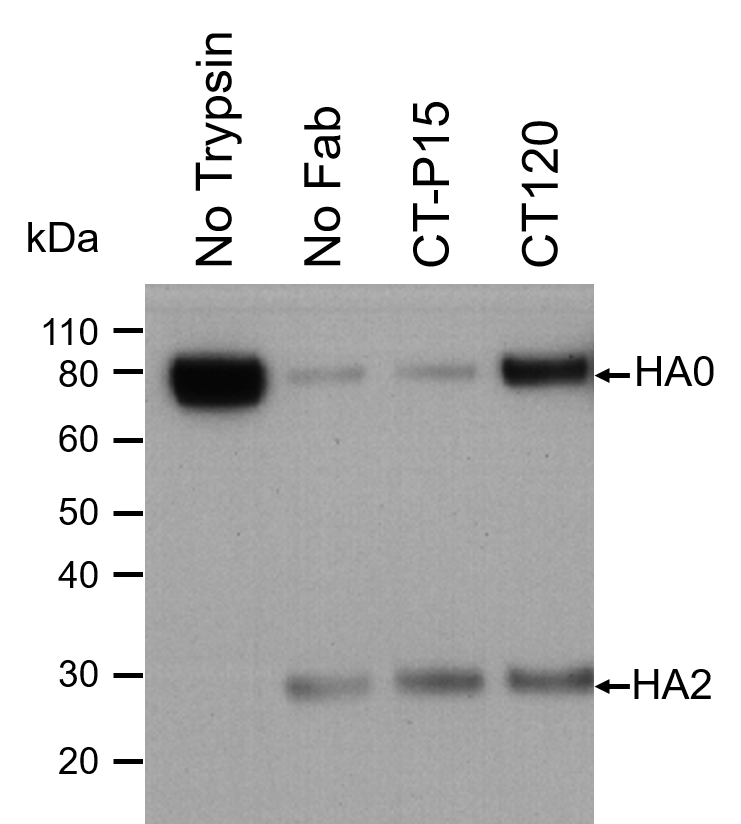

Supplement: S1 Fig — Purified HA (A/California/04/2009) and trypsin were incubated with or without Fab and subjected to SDS-PAGE. HA0 and HA2 were detected with anti-His antibody. CT120 Fab inhibited cleavage of HA0 to HA1 and HA2 by trypsin, while non-relevant Fab, CT-P15, could not. This is a representative data from 3 repeated experiments. (TIF) [file pone.0236172.s002.tif]

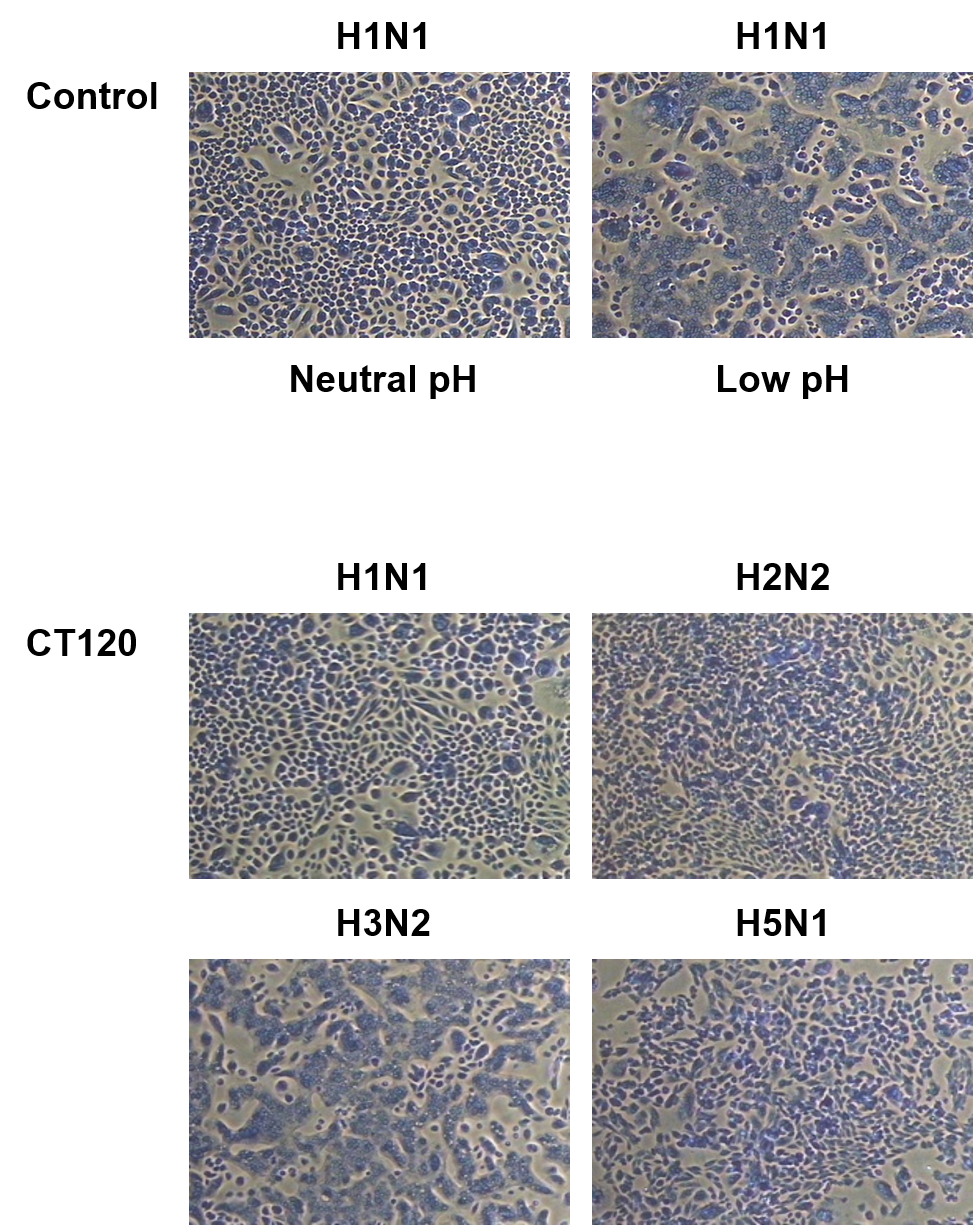

Supplement: S2 Fig — CHO cells expressing HAs from A/California/04/2009 (H1N1), A/Japan/305/1957 (H2N2), A/Brisbane/10/2007 (H3N2), and A/Vietnam/1203/2004 (H5N1) were exposed to low-pH buffer in the presence of CT149 or an isotype-matched negative control antibody (CT-P6). Representative microscope fields were captured with a digital camera using an objective (10x). This is a representative data from 3 repeated experiments. (TIF) [file pone.0236172.s003.tif]

## Slide 1
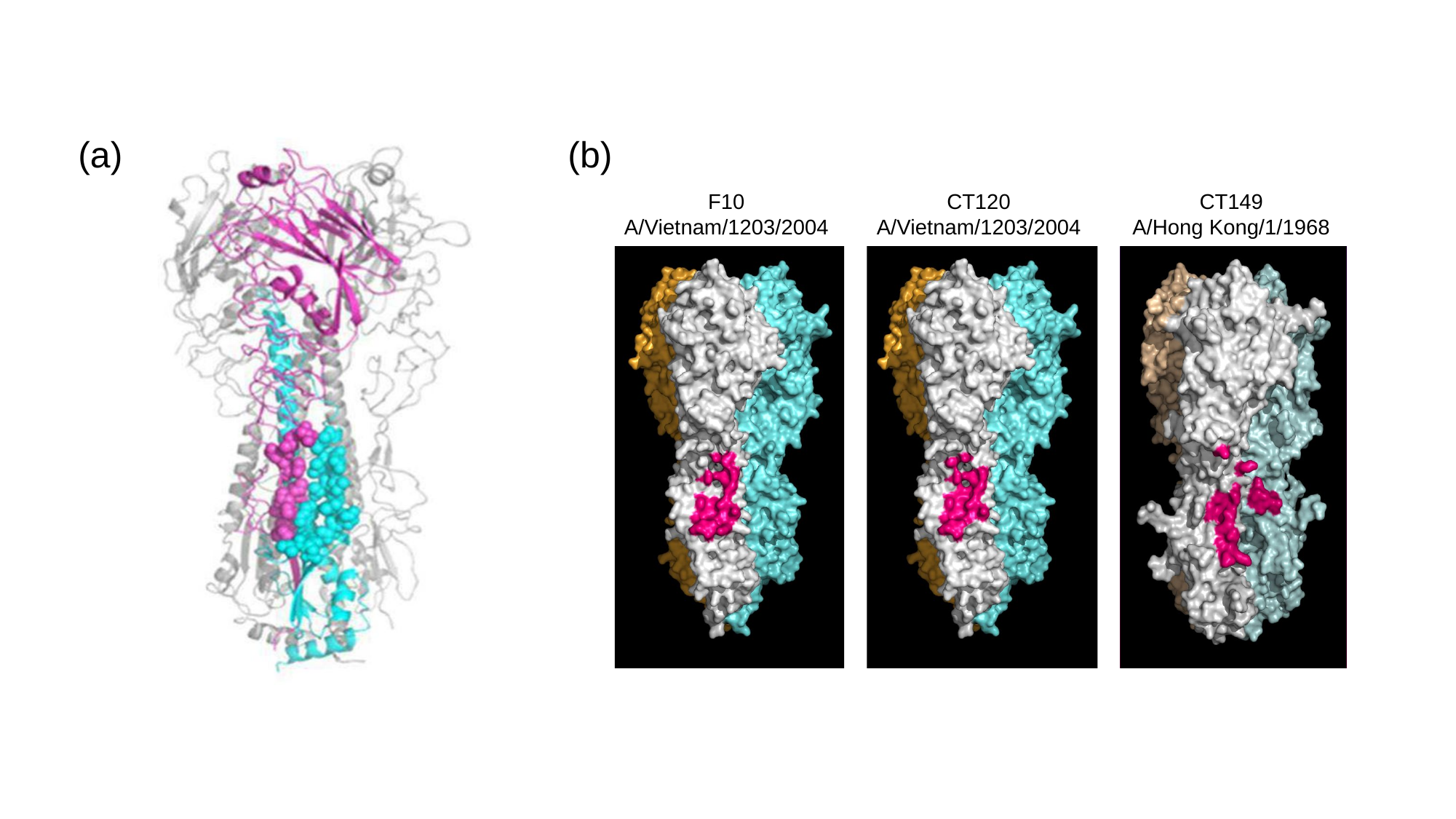

(a)
(b)
F10
A/Vietnam/1203/2004
CT120
A/Vietnam/1203/2004
CT149
A/Hong Kong/1/1968

Supplement: S3 Fig — (a) The epitope site of CT120 on H5 (A/Vietnam/1203/2004(H5N1)) is displayed in a ribbon diagram. Pink and light blue colors represent HA1 and HA2 domain respectively and gray color is neighboring monomers. Filled spaces are epitope site of CT120. It is placed on stem region. (b) Epitope sites of F10, CT120, and CT149 is marked as pink color in space-filling model of corresponding HA structure. Yellow, light blue and gray colors are each monomers of trimeric HA. (PPTX) [file pone.0236172.s004.pptx]
